# Efficacy and safety of immune checkpoint inhibitors combined with tyrosine kinase inhibitors in patients with metastatic renal cell carcinoma: a risk-stratified systematic review and meta-analysis

**DOI:** 10.3389/fimmu.2026.1805039

**Published:** 2026-05-15

**Authors:** Yingliang Rao, Jiaxiang Zhang, Xinquan Gu, Jiansong Han, Zihao Ye

**Affiliations:** 1 China-Japan Union Hospital of Jilin University, Changchun, Jilin, China; 2 Second Affiliated Hospital of Xi’an Jiaotong University, Xi’an, Shaanxi, China; 3 First Affiliated Hospital of Jilin University, Changchun, Jilin, China

**Keywords:** combination therapy, IMDC, immune checkpoint inhibitors, MRCC, tyrosine kinase inhibitors

## Abstract

**Background:**

This systematic review and meta-analysis consolidates findings from recent randomized controlled trials (RCTs) on the efficacy of first-line immune checkpoint inhibitor plus tyrosine kinase inhibitor (ICI-TKI) combination therapy for metastatic renal cell carcinoma (mRCC). We also explored prognostic biomarkers and clinical factors to inform treatment strategies.

**Methods:**

We conducted a systematic search of PubMed, Embase, the Cochrane Library, Web of Science, and major conference proceedings for RCTs published from January 2016 to December 2025. The inclusion criteria were: (1) treatment-naïve patients with mRCC; (2) intervention with first-line ICI-TKI combination therapy; and (3) availability of extractable data for survival or safety endpoints. Effect estimates were aggregated using either a random-effects model (DerSimonian–Laird) or a fixed-effects model.

**Results:**

Six RCTs were included. The primary analysis of the overall population pooled data from five trials (N = 3,637), encompassing all risk groups defined by International Metastatic Renal Cell Carcinoma Database Consortium (IMDC). Compared with control therapy, first-line ICI-TKI combination therapy significantly enhanced both progression-free survival (PFS), with a hazard ratio (HR) of 0.59 (95% confidence interval (CI) 0.51–0.67, P < 0.0001), and overall survival (OS), with an HR of 0.79 (95% CI 0.72–0.87, P < 0.0001). Efficacy varied by IMDC risk subgroup: favorable-risk patients exhibited a PFS benefit (HR 0.66, 95% CI 0.56–0.78, P < 0.0001) but no OS improvement (HR 0.96, 95% CI 0.79–1.18, P = 0.73); intermediate-risk patients had improved PFS (HR 0.60, 95% CI 0.53–0.69, P < 0.0001) and OS (HR 0.88, 95% CI 0.78–0.99, P = 0.03). Poor-risk patients derived the most substantial benefit, with improvements in both PFS (HR 0.50, 95% CI 0.41–0.61, P < 0.0001) and OS (HR 0.52, 95% CI 0.41–0.65, P < 0.0001).

**Conclusions:**

First-line combination therapy with ICI-TKI significantly improves both PFS and OS in patients with mRCC. The magnitude of clinical benefit is positively associated with higher IMDC risk scores, with the most substantial improvements in both PFS and OS observed in patients with poor-risk disease. Collectively, these findings establish ICI-TKI combination therapy as the standard first-line treatment for patients with intermediate or poor-risk mRCC.

**Systematic review registration:**

https://www.crd.york.ac.uk/PROSPERO/, identifier CRD420251272113.

## 1 Introduction

Renal cell carcinoma (RCC) constitutes roughly 90% of all primary kidney cancers. Each year, around 430,000 new cases are diagnosed worldwide, with about 20% of patients presenting with metastatic disease (mRCC) at the initial presentation. Furthermore, 20–30% of patients with localized tumors experience relapse after nephrectomy. Once distant metastases develop, the 5-year survival rate declines to below 15% (1, 2). Conventional systemic therapies, including cytokine-based regimens and agents targeting the vascular endothelial growth factor (VEGF) pathway, have shown limited clinical efficacy. Responses to these conventional therapies were frequently transient, and the rapid development of therapeutic resistance was common (3). The subsequent introduction of multi-targeted tyrosine kinase inhibitors (TKIs) improved clinical outcomes. However, durable complete responses remained rare, and acquired resistance developed in nearly all patients (4).

Immune checkpoint inhibitors (ICIs) have thus become an integral part of the treatment paradigm for mRCC (5). By blocking the programmed death 1/programmed death ligand 1 (PD-1/PD-L1) or other signaling pathways, ICIs re-energize T-cell-mediated anti-tumor immunity. When combined with anti-angiogenic TKIs, they produce complementary effects: TKIs normalize tumor vasculature, reduce immunosuppressive myeloid populations, and enhance T-cell infiltration, thereby boosting ICI activity (6, 7). Phase III trials, including KEYNOTE-426, CheckMate 9ER, and CLEAR, have demonstrated that pembrolizumab plus axitinib, nivolumab plus cabozantinib, and pembrolizumab plus lenvatinib prolong median overall survival to 45–53 months, outperforming sunitinib monotherapy and achieving objective response rates (ORR) of 55–72%. These regimens are now established as the guideline-recommended first-line standard of care for mRCC (8–10).

Accurate risk stratification is critical for guiding appropriate treatment. The Memorial Sloan Kettering Cancer Center (MSKCC) model identifies high-risk disease based on several factors: Karnofsky performance status < 80%, elevated lactate dehydrogenase or serum calcium, anemia, and a short interval from diagnosis to systemic treatment. International Metastatic RCC Database Consortium (IMDC) criteria incorporate elevated platelet and neutrophil counts and represent the most widely adopted clinical prognostic tool in contemporary practice. According to these criteria, patients are classified into three prognostic groups: favorable (no adverse factors), intermediate (1–2 factors), and poor (≥ 3 factors). This stratification correlates with a progressive decline in survival outcomes (11).

Recent landmark randomized controlled trials (RCTs)—including KEYNOTE-426, JAVELIN Renal 101, CLEAR, and CheckMate 9ER—have established the superiority of ICI-TKI combinations compared to TKI monotherapy for mRCC, demonstrating substantial improvements in progression-free survival (PFS), overall survival (OS), and ORR (12). While earlier meta-analyses have confirmed survival benefits with combination regimens, most have pooled heterogeneous interventions (e.g., ICI-TKI, ICI-ICI) and thus failed to specifically evaluate the ICI-TKI paradigm in isolation (13). Furthermore, existing syntheses offer limited granular analysis across IMDC risk strata. The most recent European Association of Urology (EAU) guidelines underscore the critical role of IMDC risk stratification in guiding first-line treatment selection; in intermediate or poor-risk disease, ICI-TKI combinations confer a significant OS advantage. Among favorable-risk patients, the survival advantage is more modest, and the magnitude of improvement over TKI monotherapy is less pronounced compared to higher-risk groups. These findings underscore that treatment response varies by prognostic risk group, informing a more personalized approach to therapy (14). However, few systematic reviews have reported stratum-specific effect estimates, limiting precision medicine decisions (15). Moreover, emerging trials conducted in Asia, such as ETER100 and RENOTORCH, have not been integrated into prior meta-analyses, leaving toxicity profiles and the durability of benefits in East Asian populations inadequately defined (16, 17). Therefore, a meta-analysis focusing solely on ICI-TKI combination therapy in mRCC, particularly one that includes an analysis of risk stratification effects, represents a valuable research direction in evidence-based medicine. Such an analysis could provide clearer evidence to support more precise treatment choices for first-line therapy in clinical practice.

This study therefore aimed at addressing these gaps through a systematic review and meta-analysis, pooling the latest landmark trial data to compare the outcomes and toxicity of first-line ICI-TKI therapy against other standards in mRCC. Specifically, we aimed to characterize efficacy differences across IMDC risk strata (favorable, intermediate, and poor), identify potential biomarkers for response, and thereby provide robust evidence to inform risk-adapted, precision first-line treatment strategies.

## 2 Methods

The conduct and reporting of this systematic review and meta-analysis strictly adhered to the Preferred Reporting Items for Systematic Reviews and Meta-Analyses (PRISMA) guidelines (18) (**Supplementary Table 1**). This meta-analysis was prospectively registered on PROSPERO (ID: CRD420251272113).

### 2.1 Search strategy

To identify relevant studies published from January 2016 to December 2025, we systematically searched PubMed, Embase, the Cochrane Library, and Web of Science. The search employed a combination of Medical Subject Headings (MeSH) terms and keywords pertaining to immune checkpoint inhibitors, tyrosine kinase inhibitors, and metastatic renal cell carcinoma. Only phase III RCTs were included. The complete search syntax is available in **Supplementary Table 2**. To capture the most recent data, we manually searched conference abstracts (2020–2023) from the major oncology congresses, including the American Society of Clinical Oncology (ASCO), European Society for Medical Oncology (ESMO), ESMO Genitourinary Cancers (ESMO GU), and American Urological Association (AUA), focusing on their annual meetings, and contacted corresponding authors by email to confirm whether trials had been fully published, thus avoiding duplication.

### 2.2 Selection criteria

After deduplication in Zotero, two reviewers (Rao and Zhang) independently and blindly assessed the titles and abstracts against the predefined PICOS framework; citations deemed uncertain at this stage were advanced to full-text assessment. The same pair then independently reviewed the full reports and cross-checked their decisions to finalize the inclusion list. Discrepancies at each stage were addressed through discussion, with a senior author (Gu) providing arbitration when necessary. The entire selection process was recorded in a PRISMA 2020 flow diagram, outlining the counts of studies identified, evaluated, and ultimately included, as well as the rationale for exclusion at each phase.

### 2.3 Data extraction

Two reviewers (Rao and Zhang) conducted data extraction independently in accordance with PRISMA guidelines. For each included trial, data were sourced from published articles, **Supplementary Materials**, clinical trial registries, and, when necessary, direct correspondence with investigators. Extracted items encompassed: (1) study characteristics (e.g., publication date, sample size, treatment regimens); (2) efficacy outcomes, including PFS, OS, ORR, and complete response (CR) rate; (3) safety outcomes, including the incidence of any-grade and grade ≥ 3 treatment-related adverse events (TRAEs); and (4) subgroup data (e.g., IMDC risk strata and PD-L1 expression level). For trials not directly reporting hazard ratios (HRs), the relevant HRs and confidence intervals (CIs) were extracted from Kaplan-Meier curves with Engauge Digitizer software (version 12.1). Consensus was achieved through discussion to resolve any discrepancies between reviewers.

### 2.4 Assessment of risk of bias

Two reviewers (Rao and Zhang) independently rated each RCT by applying the Cochrane RoB 2 tool, which assesses five domains: randomization process, deviations from intended interventions, missing outcome data, outcome measurement, and selection of reported results. Domains were categorized as “low risk,” “high risk,” or “some concerns” (19), with the judgments and supporting rationales presented in the bias assessment summary graph (**Supplementary Figure 1**).

### 2.5 Outcome measures

Primary outcomes included OS, PFS, ORR, and CR among patients with mRCC receiving combination therapy with ICI and TKI. Secondary outcomes were the incidence of TRAEs, reported as any-grade and grade ≥ 3 events. Time-to-event data are reported as HRs with 95% CIs; dichotomous outcomes were expressed as odds ratios (ORs) accompanied by 95% CIs; safety events were presented as risk ratios (RRs) alongside 95% CIs. An HR or RR < 1 and an OR > 1 consistently indicated a benefit for the ICI-TKI arm. Two reviewers independently extracted data; missing information was requested from study authors, and unavailable data were recorded as missing and excluded from the relevant analyses.

### 2.6 Statistical analysis

All statistical analyses for data synthesis and meta-analysis were performed with RevMan software (version 5.4). To evaluate statistical heterogeneity, we employed the Cochran’s Q test (χ² statistic, significance level set at P < 0.10) and quantified using the I² statistic. Thresholds around 25%, 50%, and 75% on the I² statistic were interpreted as representing low, moderate, and substantial heterogeneity, respectively. A fixed-effects model was utilized when I² < 50% and P ≥ 0.10; for all other scenarios, the DerSimonian–Laird random-effects model was employed. When substantial heterogeneity was indicated (I² ≥ 50% or P < 0.10), we conducted leave-one-out sensitivity analyses, subgroup analyses (IMDC risk and PD-L1 status), and meta-regression to investigate potential sources of heterogeneity (20, 21). To validate result stability, we systematically conducted leave-one-out sensitivity analysis, followed by meta-regression using robust variance estimation (RVE) (22).

### 2.7 Assessment of reporting bias

Due to the limited number of included RCTs (fewer than 10), funnel plots and Egger’s regression test for publication bias assessment were omitted. Therefore, potential small-study effects were assessed through qualitative discussion. We cross-verified the published results (accessed via PubMed) against the prespecified endpoints documented in the corresponding trial protocols, which were registered on ClinicalTrials.gov as well as the World Health Organization (WHO) International Clinical Trials Registry Platform (ICTRP). All predefined outcomes were fully reported, and no evidence of selective outcome reporting bias detected.

### 2.8 Evidence quality evaluation

The certainty of evidence was graded for all outcomes according to the Grading of Recommendations Assessment, Development, and Evaluation (GRADE) framework, based on evaluations across five key domains: risk of bias, inconsistency, indirectness, imprecision, and publication bias. The evidence was downgraded when applicable for any of the five domains, but no upgrading criteria were met (23). The final certainty ratings (high, moderate, low, or very low) are presented in a Summary of Findings table (**Supplementary Table 3**), which also includes absolute effect estimates and their clinical interpretations.

## 3 Results

**Figure 1:**
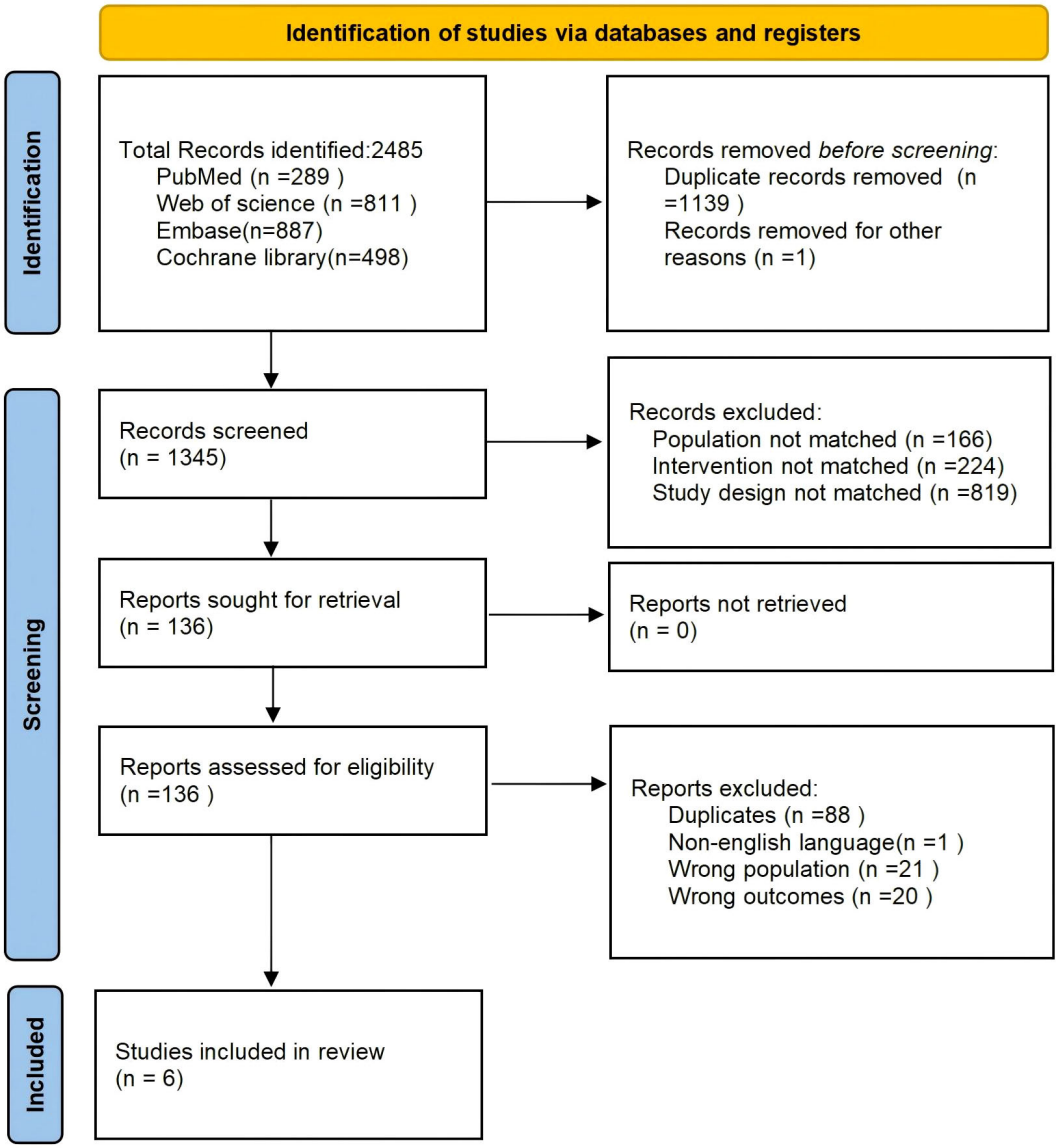
Flow diagram of literature search and study selection.

The initial search yielded 2,485 citations. Following independent dual review (Rao and Zhang) of titles and abstracts, 2,479 records were excluded. Reasons for exclusion included duplicate publications, review articles (systematic, meta-analytic, and narrative), non-randomized study designs (retrospective, single-arm, and preclinical), opinion pieces, case reports, and registered but unpublished trials. Six RCTs ultimately met the eligibility criteria (**Figure 1**). All six trials compared first-line ICI-TKI combination therapy with sunitinib monotherapy as the control. **Supplementary Table 4** presents an overview of study characteristics at baseline, while efficacy endpoints are tabulated in **Table 1**. Five trials (CheckMate 9ER, CLEAR, ETER100, KEYNOTE-426, and JAVELIN Renal 101), with an overall enrollment of 3,637 participants (1,816 allocated to ICI-TKI regimen versus 1,821 to the sunitinib arm), contributed to the primary analyses of PFS, OS, ORR, and CR. The remaining trial (RENOTORCH) enrolled exclusively patients with IMDC intermediate or poor-risk disease and, therefore, was included only in the subgroup analyses of PFS for these risk categories.

**Table 1:** Summary of primary efficacy and safety outcomes.

Study name	Treatment	Responses or complete responders/total evaluated, No. (%)		PFS		OS		TRAEs:any grade or grade≥3/total evaluated, No. (%)	
		ORR	CR	Median (95% CI), mo	HR (95% CI)	Median (95% CI), mo	HR (95% CI)	Overall	High-grade
CheckMate 9ER	N+C	180/323 (55.7%)	45/323 (13.9%)	16.4 (12.5-19.3)	0.58 (0.49-0.70)	46.5 (40.6-53.8)	0.79 (0.65-0.96)	312/320 (97.5%)	217/320 (67.8%)
	Sun	90/328 (27.4%)	15/328 (4.6%)	8.3 (7.0-9.7)		35.5 (29.2-42.8)		298/320 (93.1%)	176/320 (55.0%)
CLEAR	P+L	253/355 (71.3%)	65/355 (18.3%)	23.9 (20.8-27.7)	0.47 (0.38-0.57)	53.7 (48.7-NE)	0.79 (0.63-0.99)	341/352 (96.9%)	261/352 (74.1%)
	Sun	131/357 (36.7%)	17/357 (4.8%)	9.2 (6.0-11.0)		54.3 (40.9-NE)		313/340 (92.1%)	205/340 (60.3%)
ETER100	B+A	189/264 (71.6%)	3/264 (0.8%)	19.0 (15.3-22.8)	0.53 (0.42-0.67)	NE	0.66 (0.48-0.92)	262/264 (99.2%)	178/264 (67.4%)
	Sun	66/263 (25.1%)	0/263 (0%)	9.8 (8.4-12.4)		NE		262/264 (99.2%)	174/263 (66.2%)
JAVELIN	A+A	264/442 (59.7%)	25/442 (5.7%)	13.9 (11.1-16.6)	0.66 (0.57-0.77)	44.8 (39.7-51.1)	0.88 (0.75-1.04)	420/434 (96.8%)	290/434 (66.8%)
	Sun	142/444 (32.0%)	16/444 (3.6%)	8.5 (8.2-9.7)		38.9 (31.4-45.2)		425/439 (96.8%)	270/439 (61.5%)
KEYNOTE-426	P+A	262/432 (55.7%)	50/432 (11.6%)	15.7 (13.6-20.2)	0.69 (0.59-0.81)	47.2 (43.6-54.8)	0.84 (0.71-0.99)	421/429 (98.1%)	326/429 (76.0%)
	Sun	170/429 (27.4%)	17/429 (4.0%)	11.1 (8.9-12.5)		40.8 (34.3-47.5)		408/425 (96.0%)	306/425 (72.0%)
RENOTORCH	T+A	119/210 (56.7%)	10/210 (4.8%)	18.0 (15.0-NE)	0.65 (0.49-0.86)	NE	0.61 (0.40-0.92)	207/208 (99.5%)	148/208 (71.2%)
	Sun	65/211 (30.8%)	8/211 (3.8%)	9.8 (8.3-13.8)		26.8 (NR)		209/210 (99.5%)	141/210 (67.1%)

### 3.1 Progression-free survival

**Figure 2:**
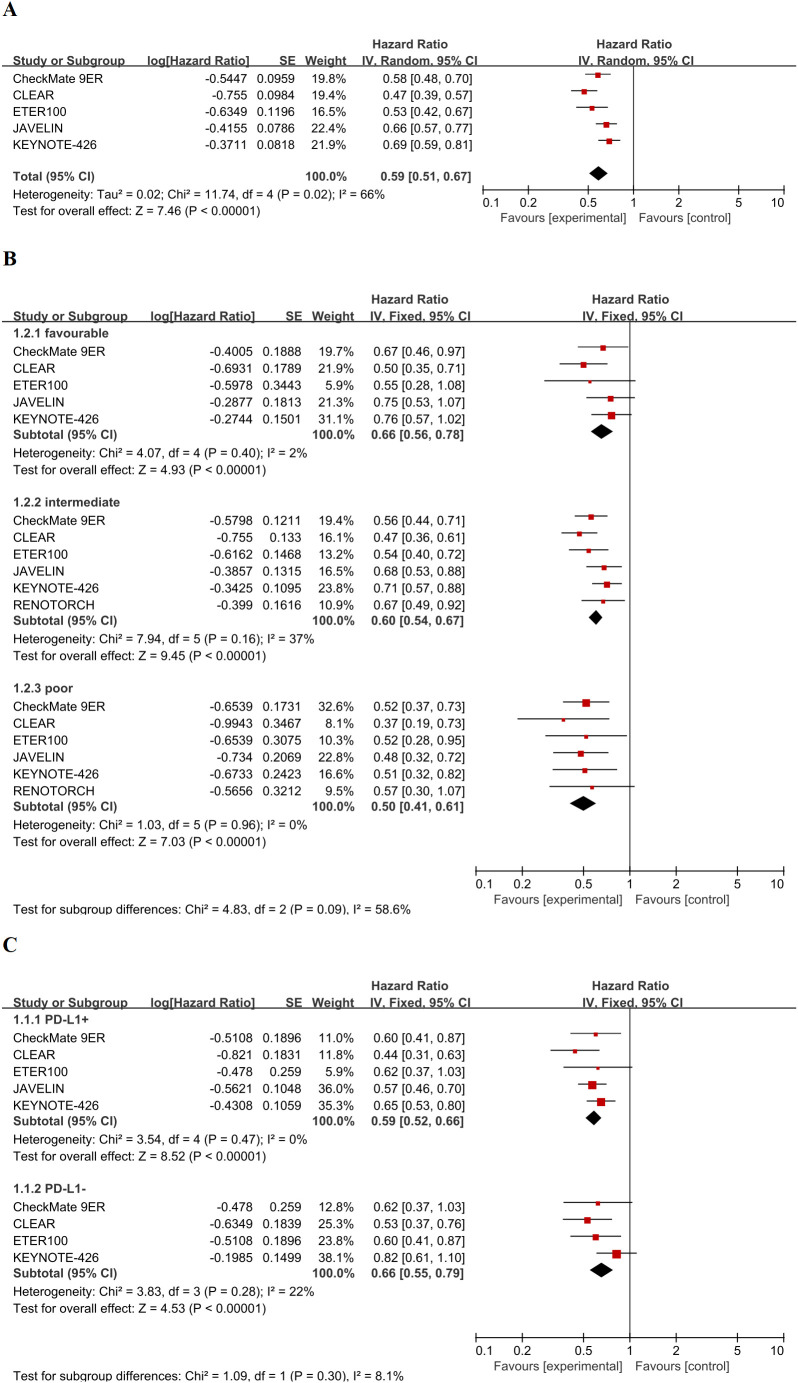
Forest plots of progression-free survival. **(A)** Random-effects model forest plot of the overall pooled analysis comparing first-line immune checkpoint inhibitor plus tyrosine kinase inhibitor (ICI-TKI) combination therapy versus sunitinib. **(B)** Fixed-effects model forest plots of PFS stratified by IMDC risk criteria (favorable, intermediate, and poor risk). The test for subgroup differences is reported below the plots. **(C)** Fixed-effects model forest plot of PFS stratified by tumor PD-L1 expression status (≥1% vs. <1%).

For the primary analysis, pooling data from five trials with broad inclusion criteria (overall population), the HR for PFS was assessed for the ICI-TKI combination regimen versus the sunitinib control. The forest plot (**Figure 2A**) showed that the point estimates and 95% CIs for all five trials were located entirely to the left of the line of no effect (HR = 1), indicating a consistent treatment benefit favoring ICI-TKI therapy across studies. Consequently, a random-effects meta-analysis confirmed a significant PFS benefit for ICI-TKI therapy, yielding a pooled HR of 0.59 (95% CI 0.51–0.67, P < 0.0001). This corresponds to a 41% reduction in the risk of disease progression or death. Substantial heterogeneity was observed among the studies (τ² = 0.02; χ² = 11.74, df = 4, P = 0.02; I² = 66%). The robustness of this primary finding was confirmed through sensitivity analyses, including leave-one-out analysis and meta-regression with RVE. The pooled treatment effect remained statistically significant in all sensitivity analyses.

### 3.2 Progression-free survival according to subgroups

To account for observed heterogeneity, we undertook subgroup analyses prespecified according to IMDC risk classification (**Figure 2B**). Five trials contributed data to the favorable-risk stratum. The intermediate and poor-risk strata additionally included the RENOTORCH trial, which enrolled exclusively intermediate or poor-risk patients. Fixed-effects models demonstrated significant PFS benefits across all strata: favorable-risk (HR 0.66, 95% CI 0.56–0.78, P < 0.0001), intermediate-risk (HR 0.60, 95% CI 0.53–0.69, P < 0.0001), and poor-risk (HR 0.50, 95% CI 0.41–0.61, P < 0.0001). Heterogeneity within each stratum was low (favorable-risk: I² = 2%, P = 0.40; intermediate-risk: I² = 37%, P = 0.16; poor-risk: I² = 0%, P = 0.96), supporting the use of IMDC risk categories to explain inter-study variance. Sensitivity analyses that excluded RENOTORCH yielded the following results for the intermediate-risk stratum: HR 0.59 (95% CI 0.51–0.69, P < 0.0001); χ² decreased from 7.94 to 7.46, and I² increased from 37% to 46%. For the poor-risk stratum: HR 0.49 (95% CI 0.40–0.60, P < 0.0001); χ² decreased from 1.03 to 0.85, and I² remained at 0%. These sensitivity analyses confirmed that the inclusion of RENOTORCH did not materially alter the stratum-specific estimates, as the magnitude of benefit, statistical significance, and heterogeneity levels remained consistent. The test for subgroup differences (interaction) showed a non-significant trend (χ² = 4.73, df = 2, P = 0.09; I² = 57.7%), suggesting that PFS benefit might be greater with higher IMDC risk, although this did not reach conventional statistical significance.

We further analyzed PFS stratified by tumor PD-L1 expression level (**Figure 2C**). Among patients with PD-L1 expression ≥ 1%, ICI-TKI therapy was associated with a significant 41% decrease in progression risk (HR 0.59, 95% CI 0.52–0.66, P < 0.0001). In patients with PD-L1 expression < 1%, ICI-TKI therapy demonstrated a 34% reduction in risk (HR 0.66, 95% CI 0.55–0.76, P < 0.0001). Both subgroups exhibited minimal heterogeneity (I² = 0% for PD-L1 ≥ 1% and I² = 22% for PD-L1 < 1%). The JAVELIN Renal 101 trial was excluded from the PD-L1 < 1% analysis because it did not report data for this specific subgroup. No significant interaction was observed between the PD-L1 subgroups (χ² = 1.09, df = 1, P = 0.30; I² = 8.1%), suggesting a consistent treatment effect across PD-L1 expression levels, and indicating that PD-L1 expression status did not significantly modify the PFS benefit of ICI-TKI combination therapy.

### 3.3 Overall survival

**Figure 3:**
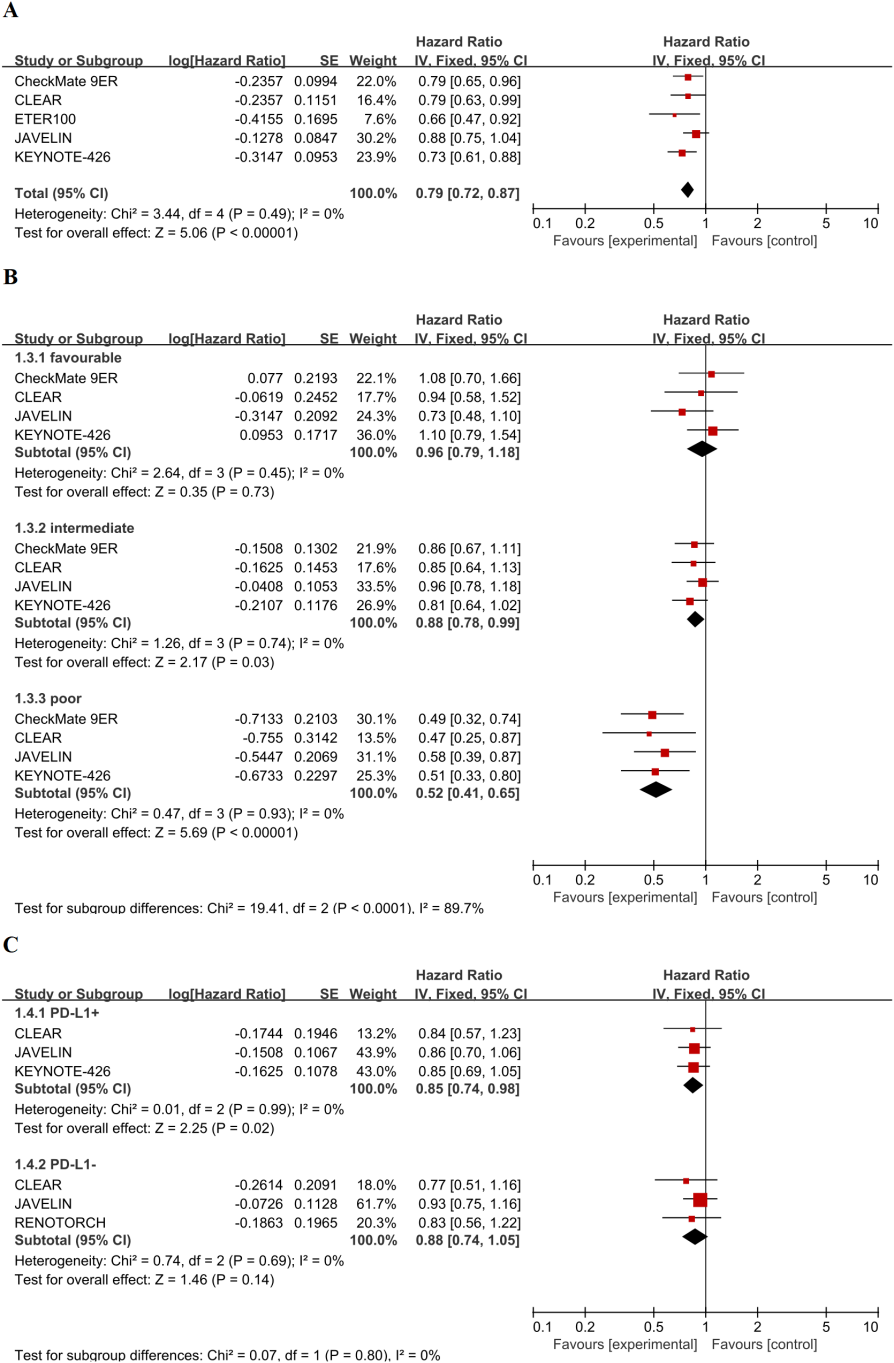
Forest plots of overall survival. **(A)** Fixed-effects model forest plot of the overall pooled analysis comparing first-line ICI-TKI combination therapy versus sunitinib. **(B)** Fixed-effects model forest plots of OS stratified by IMDC risk criteria (favorable, intermediate, and poor risk). The test for subgroup differences is reported below the plots. **(C)** Fixed-effects model forest plot of OS stratified by tumor PD-L1 expression status (≥1% vs. <1%).

The forest plot (**Figure 3A**) showed that four trials demonstrated a statistically significant OS benefit (with point estimates lying to the left of the line of no effect), while one trial had a 95% CI that crossed this line. A fixed-effects meta-analysis confirmed a significant OS benefit for ICI-TKI therapy, with a pooled HR of 0.79 (95% CI 0.72–0.87, P < 0.0001). This corresponds to a 21% reduction in the risk of death. No significant heterogeneity was observed among the trials (χ² = 3.44, I² = 0%). The robustness of this OS benefit was further supported by sensitivity analyses, including leave-one-out analysis and meta-regression with robust variance estimation.

### 3.4 Overall survival according to subgroups

We analyzed OS based on IMDC risk strata (**Figure 3B**). Because the ETER100 trial did not report OS data by IMDC subgroup, only four trials contributed to this analysis. Fixed-effects modeling showed no significant OS benefit for ICI-TKI therapy in the favorable-risk stratum (HR 0.96, 95% CI 0.79–1.18, P = 0.73), with no heterogeneity observed (I² = 0%, P = 0.45). In contrast, both intermediate-risk (HR 0.88, 95% CI 0.78–0.99, P = 0.03) and poor-risk patients (HR 0.52, 95% CI 0.41–0.65, P < 0.0001) saw significant OS benefits. Heterogeneity was negligible in both strata (I² = 0%, with P = 0.74 and P = 0.93, respectively). The test for subgroup interaction across IMDC risk strata was statistically significant (χ² = 19.41, df = 2, P < 0.0001; I² = 89.7%), confirming that the magnitude of OS benefit from ICI-TKI therapy increases with higher IMDC risk.

For the analysis of OS by PD-L1 expression, only three of the five eligible RCTs could be included, as CheckMate 9ER and ETER100 did not report OS outcomes stratified by PD-L1 status (**Figure 3C**). In the PD-L1-positive subgroup (expression ≥ 1%), ICI-TKI therapy was associated with a significant 15% reduction in mortality risk (HR 0.85, 95% CI 0.74–0.98, P = 0.02). In the PD-L1-negative subgroup (< 1%), the point estimate favored ICI-TKI but was not statistically significant (HR 0.88, 95% CI 0.74–1.05, P = 0.14). No heterogeneity was detected in either PD-L1 subgroup (I² = 0%). The test for interaction between PD-L1 subgroups was not statistically significant (χ² = 0.07, df = 1, P = 0.80; I² = 0%), indicating that PD-L1 expression level did not significantly modify the OS benefit of ICI-TKI therapy.

### 3.5 Objective response rate and complete response

**Figure 4:**
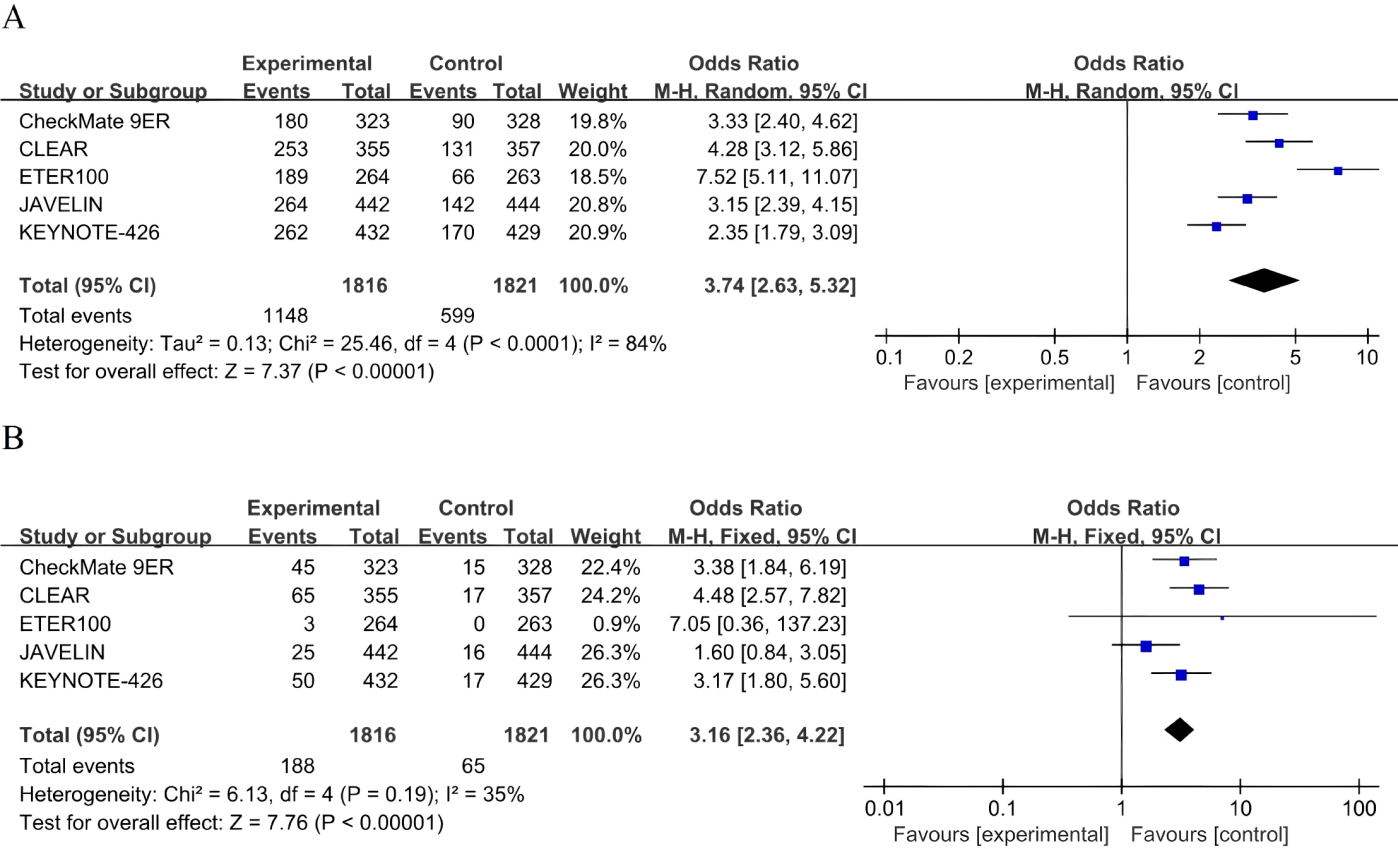
Forest plots of tumor response outcomes. **(A)** Random-effects model forest plot of objective response rate (ORR). **(B)** Fixed-effects model forest plot of complete response (CR) rate. All effect estimates lie in the OR > 1 region, indicating superior ORR and CR with ICI-TKI compared with sunitinib.

Across five RCTs (n = 3,637), ICI-TKI showed a significant advantage in ORR over sunitinib (pooled odds ratio [OR] 3.74, 95% CI 2.63–5.32, P < 0.0001) (**Figure 4A**). Due to considerable inconsistency among studies (I² = 84%, P < 0.0001), a random-effects model was applied. Sensitivity analyses identified ETER100 and KEYNOTE-426 as the main contributors to heterogeneity. Excluding these two trials reduced the I² statistic to 9% (**Supplementary Figure 2**), suggesting that differences in the specific tyrosine kinase inhibitors used and in response assessment criteria were the primary drivers of the observed variability.

Among the same patient population (n = 3,637), the CR rate significantly favored ICI-TKI therapy over sunitinib (OR 3.16, 95% CI 2.36–4.22, P < 0.0001) (**Figure 4B**). Given the moderate heterogeneity observed (I² = 31%), a fixed-effects model was applied.

### 3.6 Treatment-related adverse events

**Figure 5:**
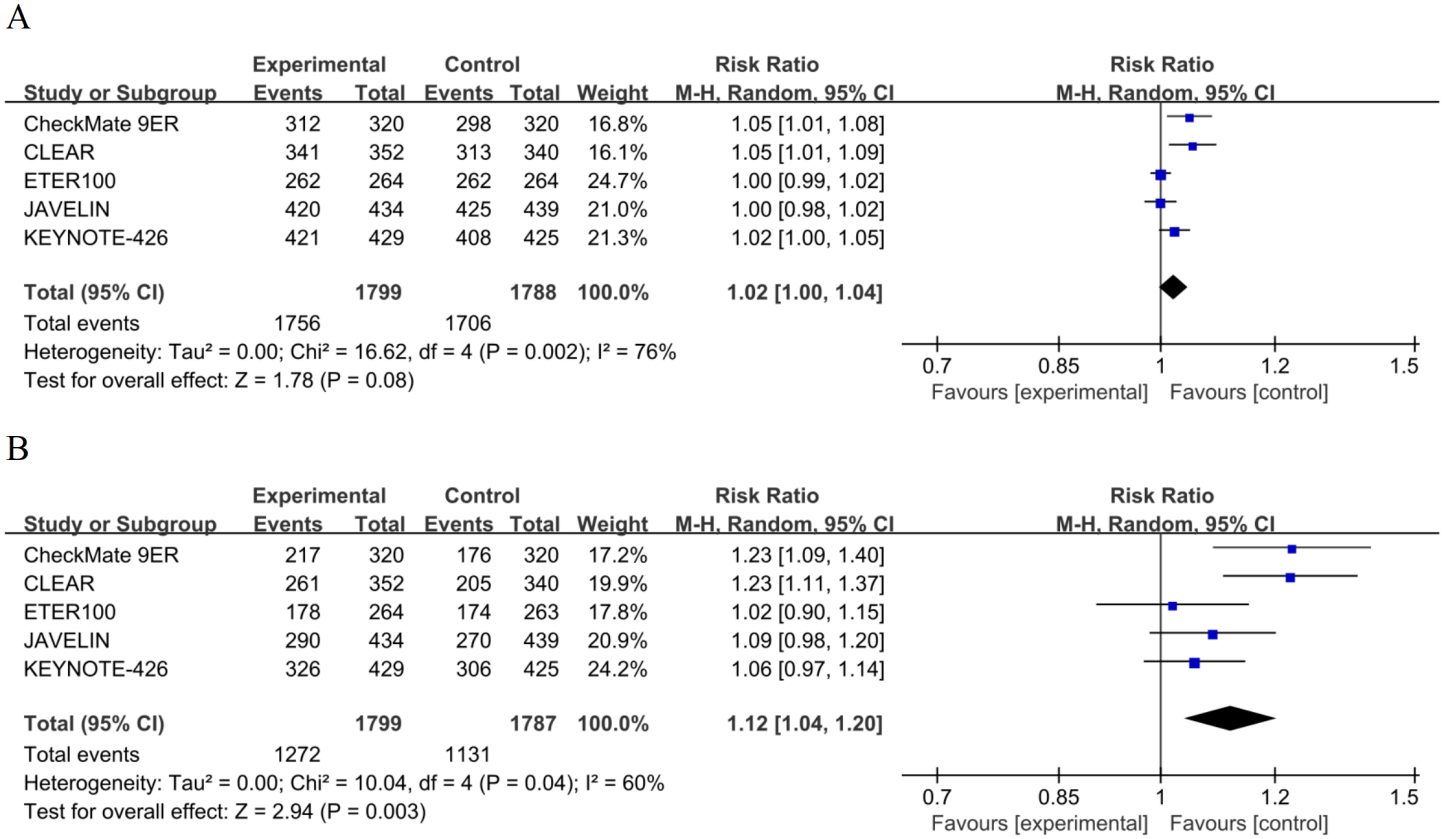
Forest plots of treatment-related adverse events. **(A)** Random-effects model forest plot of any-grade TRAEs. **(B)** Random-effects model forest plot of grade ≥3 TRAEs.

Five RCTs (n = 3,587) reported any-grade TRAEs. Among 1,799 patients receiving ICI-TKI, 1,756 (97.6%) experienced any-grade TRAEs, compared with 1,706 of 1,788 (95.4%) in the sunitinib arm. The combined RR was 1.02 (95% CI 1.00–1.04, P = 0.08, random-effects model), indicating no significant difference (**Figure 5A****).** Moderate heterogeneity was observed (I² = 76%, P = 0.002). Leave-one-out analysis showed that simultaneous exclusion of ETER100 (multi-targeted TKI) and JAVELIN Renal 101 (PD-L1-based combination) reduced I² to 20%, with RR remaining approximately 1.00 (**Supplementary Figure 3****).** Overall, ICI-TKI did not meaningfully increase the risk of any-grade TRAEs.

For grade ≥ 3 TRAEs, ICI-TKI (1,272 of 1,799, 70.7%) showed a significantly greater incidence versus sunitinib (1,131 of 1,787, 63.3%); pooled RR 1.12 (95% CI 1.04–1.20, P = 0.003, random-effects model). Moderate between-study inconsistency was observed (I² = 60%, P = 0.04) (**Figure 5B****).** Excluding CLEAR and CheckMate 9ER reduced I² to 0% and RR remained 1.10, suggesting that potent vascular endothelial growth factor receptor-tyrosine kinase inhibitor (VEGFR-TKI) plus PD-1 blockade may be a key contributor to this variability (**Supplementary Figure 4**). Overall, ICI-TKI increases grade ≥ 3 toxicity by approximately 12% while improving efficacy, necessitating a careful benefit-risk assessment in clinical practice.

### 3.7 Risk of bias and certainty of evidence

The assessment of study bias, based on the RoB 2 tool, is detailed in **Supplementary Table 1**. All trials were assessed as having a low risk of bias in the randomization process. Incomplete blinding led to a rating of “some concerns” for deviations from intended interventions in three trials; all other domains were rated as low risk. Consequently, the overall risk of bias was considered low for all included trials. Comparison of the published results with the registered protocols confirmed that all prespecified primary and secondary outcomes were fully reported for the six included trials, with no evidence of selective outcome reporting. Owing to the limited number of included trials (< 10), funnel plots or Egger’s test could not be performed to formally assess publication bias. In accordance with GRADE guidance, the publication bias domain was therefore rated as “undetected.” The Summary of Findings table (**Supplementary Table 2**) provides high-certainty evidence for the four patient-important outcomes: PFS, OS, ORR, and grade ≥ 3 TRAEs. Therefore, the pooled estimates presented in this meta-analysis provide a reliable reflection of the true treatment effects of first-line ICI-TKI combination therapy compared with sunitinib monotherapy in treatment-naïve adults with mRCC.

## 4 Discussion

Six distinct ICI-TKI regimens have expanded first-line treatment options for mRCC, each demonstrating a long-term survival plateau. However, their toxicity profiles differ, and their efficacy varies according to IMDC risk stratification and PD-L1 expression, necessitating a careful balance between efficacy, safety, and biomarker status in clinical decision-making. The absence of head-to-head trials necessitates indirect comparisons to evaluate survival differences. Using sunitinib as a common comparator, our meta-analysis demonstrates that ICI-TKI combination therapy reduces the risk of progression by 41% (PFS HR 0.59, 95% CI 0.51–0.67) and the risk of death by 21% (OS HR 0.79, 95% CI 0.72–0.87). This provides high-certainty evidence to support risk-adapted first-line treatment selection.

Focusing on IMDC risk strata, patients with favorable-risk disease have not demonstrated a clear overall survival benefit from ICI-TKI therapy. A recent meta-analysis of all ICI-based combinations (Tucci et al.) found no OS advantage for favorable-risk patients (HR 0.99, 95% CI 0.79–1.25, P = 0.96), whereas significant benefits were noted for intermediate-risk (HR 0.68) and poor-risk patients (HR 0.49), with a significant test for interaction (P < 0.001). For PFS, the benefit in favorable-risk disease was marginal and non-significant (HR 0.79, 95% CI 0.51–1.21). In contrast, intermediate-risk and poor-risk patients exhibited clear and progressively greater PFS improvements (HR 0.64 and 0.50, respectively), with a significant interaction test (P < 0.001). Although the control arms in the analysis by Tucci et al. included various ICI-based combinations, the observed gradient of treatment effect across IMDC risk strata still reflects the differential benefit derived from ICI-TKI therapy, thereby corroborating our findings (13). Our global cohort confirms improved PFS and OS with ICI-TKI. Subgroup tests show a gradient PFS benefit (favorable, intermediate, poor-risk all significant), while OS significance is confined to intermediate or poor-risk patients. Consequently, although current international guidelines endorse immune-oncology regimens as the default first-line approach following “risk equalization,” those with favorable-risk disease still require a careful trade-off between postponing resistance and incurring chronic immunotoxicity. A rational, evidence-based strategy is initial TKI monotherapy with early, closely monitored transition to sequential immunotherapy upon progression, thereby maximizing the likelihood of durable survival while preserving quality of life (24). For intermediate and poor-risk patients, this meta-analysis provides high-certainty evidence that ICI-TKI significantly prolongs PFS and confers clear survival advantage.

Consistent with previous reports, our analysis indicates that PD-L1 status has limited predictive value for benefiting from ICI-TKI, with significant PFS and OS gains observed irrespective of PD-L1 status. Future research should focus on developing multiparameter predictive models. Analyses of biospecimens from the JAVELIN Renal 101 trial (Choueiri et al.) demonstrated that baseline blood neutrophil and platelet counts, along with systemic inflammatory indices, predicted benefit from TKI monotherapy. In contrast, immunotherapy-based combinations were associated with enrichment of innate immune cell subsets and increased T-cell receptor diversity (25, 26). These findings suggest that dynamic, integrated “blood-tumor” immune profiling could potentially supplant single-marker PD-L1 testing and inform more personalized treatment sequencing or combination strategies (27).

Pooled data demonstrate that ICI-TKI combination therapy yields significantly higher rates of both ORR (OR 3.74, 95% CI 2.63–5.32, P < 0.0001) and CR (OR 3.16, 95% CI 2.36–4.22, P < 0.0001) compared with TKI monotherapy, indicating deeper tumor regression in a greater proportion of patients. Based on the pooled objective response rate (63% versus 33%), the number needed to treat (NNT) to achieve benefit is approximately 3, meaning that for every three patients treated, one additional response is gained. Given the lowest HR for OS in the poor-risk subgroup (0.52), the NNT in this population is expected to be even more favorable; however, precise calculation of stratum-specific NNTs awaits validation through individual patient data analysis. Although substantial heterogeneity was observed for ORR (I² = 84%), a sensitivity analysis excluding the ETER100 (featuring a multi-targeted TKI) and KEYNOTE-426 (axitinib-pembrolizumab) trials reduced the I² statistic to 9%, while the treatment effect remained substantial (OR 3.52, 95% CI 2.93–4.24). This suggests that the observed variability was driven primarily by differences in the specific VEGFR-TKI used, imaging assessment schedules, and response thresholds, rather than by a loss of intrinsic efficacy of the ICI-TKI backbone. Heterogeneity for CR was consistently low across studies (I² = 31%), confirming the robustness of this deep anti-tumor response. From a clinical perspective, patients who achieve a CR frequently enter a subsequent survival plateau. This provides a critical window for considering local consolidative therapies or a treatment pause, representing a potential step toward a “functional cure” (28).

Regarding safety, any-grade TRAEs were comparable between treatment arms (RR 1.02, 95% CI 1.00–1.04, P = 0.08), while grade ≥ 3 TRAEs were modestly higher with the combination (RR 1.12, 95% CI 1.04–1.20). The absolute excess is manageable in routine clinical practice. Notably, grade ≥ 3 TRAEs in ICI-TKI therapy may be irreversible, including hypothyroidism (requiring lifelong levothyroxine replacement), hypophysitis/adrenal insufficiency (requiring lifelong glucocorticoid replacement), and type 1 diabetes mellitus (requiring lifelong insulin therapy), whereas TKI-related toxicities (hypertension, proteinuria, hand-foot syndrome, diarrhea) are typically reversible upon drug discontinuation or dose modification. Therefore, clinical decision-making should consider not only the incidence but also the reversibility of toxicities and their long-term impact on quality of life. Patients with high-risk or bulky disease requiring rapid tumor shrinkage can still receive the combination first-line, provided that blood pressure, liver enzymes, and diarrhea are monitored and stepwise dose modifications are planned. For older patients or those with multiple underlying health conditions, a TKI with a milder toxicity profile or a dual-immunotherapy backbone may be chosen to individualize the benefit-risk balance (29). Leave-one-out analyses showed that heterogeneity for any-grade TRAEs was mainly driven by ETER100 and JAVELIN Renal 101 (avelumab-axitinib); removing them reduced I² from 76% to 20%. Heterogeneity for grade ≥ 3 TRAEs was attributable to potent VEGFR-TKI studies; after their exclusion, I² fell to 0%, while the effect estimate remained stable.

Our review has several strengths. It is the first systematic appraisal focused specifically on first-line ICI-TKI combinations, includes six phase III RCTs (incorporating the latest Chinese data), and emphasizes subgroup analysis by IMDC risk. Rigorous statistical methods were applied, and no publication bias was detected, providing the most up-to-date evidence base for clinicians. Limitations arise from the aggregate nature of the data: patient-level confounders (age, comorbidities, metastatic sites) could not be adjusted for, and differences in follow-up duration and endpoint adjudication may introduce measurement bias. Despite random-effects modeling and extensive sensitivity analyses, significant heterogeneity persisted for ORR and TRAEs; these pooled estimates should therefore be interpreted cautiously alongside individual patient characteristics. Looking ahead, the field of first-line ICI-TKI therapy for mRCC continues to evolve rapidly. Currently, several phase III RCTs are underway, broadly encompassing three directions: (1) evaluation of novel ICI-TKI combinations, such as tislelizumab plus lenvatinib; (2) head-to-head comparisons of existing ICI-TKI regimens to clarify efficacy and safety differences between specific combinations; and (3) exploration of predictive biomarker-driven strategies for individualized treatment selection. These studies are expected to address critical unanswered questions within the next 3–5 years and further optimize the first-line treatment landscape. However, based on the findings of this meta-analysis and remaining evidence gaps, we propose that future research should prioritize two key areas: first, conducting head-to-head trials comparing “TKI monotherapy followed by sequential ICI” versus upfront “ICI-TKI combination” in IMDC favorable-risk patients, to verify whether deferred immunotherapy maintains efficacy while reducing toxicity; and second, developing and validating predictive biomarkers that can guide individualized treatment decisions, thereby translating risk-stratified therapy into true precision medicine.

## 5 Conclusion

This meta-analysis provides high-certainty evidence that first-line ICI-TKI combination therapy significantly prolongs both progression-free and overall survival in patients with mRCC, with the magnitude of benefit increasing across higher IMDC risk categories. The most pronounced efficacy was observed in the intermediate and poor-risk subgroups. In contrast, patients with favorable-risk disease require individualized benefit–risk assessment. Collectively, these findings provide a robust, evidence-based justification for implementing risk-adapted treatment strategies and establish a foundation for future biomarker-driven precision oncology trials.

## Data Availability

The original contributions presented in the study are included in the article/**Supplementary Material**. Further inquiries can be directed to the corresponding author.
