# Supplementary material for: Efficacy and safety of immune checkpoint inhibitors combined with tyrosine kinase inhibitors in patients with metastatic renal cell carcinoma: a risk-stratified systematic review and meta-analysis
Source: Front Immunol. 2026 May 15;17:1805039. doi: 10.3389/fimmu.2026.1805039 (PMC13220056; doi:10.3389/fimmu.2026.1805039)
Supplement: Supplementary file 1 [file Table1.docx]

**Supplementary Table 1: Checklist of items for reporting a systematic review and meta-analysis**

| **Section and topic** | **Item No** | **Checklist item** | **Reported on page No** |
| --- | --- | --- | --- |
| **Title** |  |  |  |
| Title | 1 | Identify the report as a systematic review, meta-analysis, or both | 1 |
| **Abstract** |  |  |  |
| Structured summary | 2 | Provide a structured summary including, as applicable, background, objectives,data sources, study eligibility criteria, participants, interventions, study appraisaland synthesis methods, results, limitations, conclusions and implications of key findings, systematic review registration number | 1-2 |
| **Introduction** |  |  |  |
| Rationale | 3 | Describe the rationale for the review in the context of what is already known | 2-3 |
| Objectives | 4 | Provide an explicit statement of the objective(s) or question(s) the review addresses. | 3 |
| **Methods** |  |  |  |
| Protocol and registration | 5 | Indicate if a review protocol exists, if and where it can be accessed (such as web address), and, if available, provide registration information including registration number | 4 |
| Eligibility criteria | 6 | Specify the inclusion and exclusion criteria for the review and how studies were grouped for the syntheses. | 4 |
| Information sources | 7 | Specify all databases, registers, websites, organisations, reference lists and other sources searched or consulted to identify studies.  Specify the date when each source was last searched or consulted. | 4 |
| Search strategy | 8 | Present the full search strategies for all databases, registers and websites, including any filters and limits used. | 4 |
| Selection process | 9 | Specify the methods used to decide whether a study met the inclusion criteria of the review, including how many reviewers screened each  record and each report retrieved, whether they worked independently, and if applicable, details of automation tools used in the process. | 4 |
| Data collection process | 10 | Specify the methods used to collect data from reports, including how many reviewers collected data from each report, whether they worked  independently, any processes for obtaining or confirming data from study investigators, and if applicable, details of automation tools used in  the process. | 4 |
| Data items | 11 | List and define all variables for which data were sought (such as PICOS, funding sources) and any assumptions and simplifications made | 4 |
| Study risk of bias  assessment | 12 | Specify the methods used to assess risk of bias in the included studies, including details of the tool(s) used, how many reviewers assessed  each study and whether they worked independently, and if applicable, details of automation tools used in the process. | 4 |
| Effect measures | 13 | Specify for each outcome the effect measure(s) (e.g. risk ratio, mean difference) used in the synthesis or presentation of results. | 5 |
| Synthesis methods | 14 | Describe the methods of handling data and combining results of studies, if done, including measures of consistency (such as I2 statistic) for each meta analysis | 5 |
| Reporting bias  assessment | 15 | Describe any methods used to assess risk of bias due to missing results in a synthesis (arising from reporting biases). | 5 |
| Certainty assessment | 16 | Describe any methods used to assess certainty (or confidence) in the body of evidence for an outcome. | 5 |
| **Results** |  |  |  |
| Study selection | 17 | Give numbers of studies screened, assessed for eligibility, and included in the review, with reasons for exclusions at each stage, ideally with a flow diagram | 6 |
| Study characteristics | 18 | Cite each included study and present its characteristics. | 6 |
| Risk of bias in studies | 19 | Present assessments of risk of bias for each included study. | 8 |
| Results of individual studies | 20 | For all outcomes considered (benefits or harms), present for each study (a) simple summary data for each intervention group and (b) effect estimates and confidence intervals, ideally with a forest plot | 6-8 |
| Results of syntheses | 21 | Present results of each meta-analysis done, including confidence intervals and measures of consistency | 6-8 |
| Reporting biases | 22 | Present assessments of risk of bias due to missing results (arising from reporting biases) for each synthesis assessed. | 8 |
| Certainty of evidence | 23 | Present assessments of certainty (or confidence) in the body of evidence for each outcome assessed. | 6-8 |
| **Discussion** |  |  |  |
| Summary of evidence | 24 | Summarise the main findings including the strength of evidence for each main outcome; consider their relevance to key groups (such as health care providers, users, and policy makers) | 9-10 |
| Limitations | 25 | Discuss limitations at study and outcome level (such as risk of bias), and at review level (such as incomplete retrieval of identified research, reporting bias) | 10 |
| Conclusions | 26 | Provide a general interpretation of the results in the context of other evidence, and implications for future research | 10-11 |
| **Funding** |  |  |  |
| Funding | 27 | Describe sources of funding for the systematic review and other support (such as supply of data) and role of funders for the systematic review | NA |

**Supplementary Table 2: Searches performed in Pubmed, Embase, the Cochrane Library and Web of Science**

| ***PubMed*** | | |
| --- | --- | --- |
| ***#1*** | (renal cell carcinoma[MeSH Terms]) OR (renal cell carcinoma[Title/Abstract]) OR (kidney cancer[Title/Abstract]) OR (metastatic renal cell carcinoma[Title/Abstract]) OR (mRCC[Title/Abstract]) | ***32194*** |
| ***#2*** | (immune checkpoint inhibitor[MeSH Terms]) OR (nivolumab[Title/Abstract]) OR (pembrolizumab[Title/Abstract]) OR (ipilimumab[Title/Abstract]) OR (avelumab[Title/Abstract]) OR (atezolizumab[Title/Abstract]) OR(toripalimab[Title/Abstract]) | ***48884*** |
| ***#3*** | (tyrosine kinase inhibitor[MeSH Terms]) OR (TKI[Title/Abstract]) OR (axitinib[Title/Abstract]) OR (cabozantinib[Title/Abstract]) OR (lenvatinib[Title/Abstract]) OR (sunitinib[Title/Abstract]) OR (anlotinib[Title/Abstract]) | ***21689*** |
| ***#4*** | (randomized[Title/Abstract]) OR (randomised[Title/Abstract]) OR ("phase III"[Title/Abstract]) OR ("phase 3"[Title/Abstract]) OR (RCT[Title/Abstract]) | ***537463*** |
| ***#5*** | *#1 AND #2 AND #3 AND #4* | ***Final search***  ***289*** |
| ***Embase*** | | |
| ***#1*** | 'renal cell carcinoma':ab,ti OR 'kidney cancer':ab,ti OR 'metastatic renal cell carcinoma':ab,ti OR 'mRCC':ab,ti | ***85353*** |
| ***#2*** | 'immune checkpoint inhibitor':ab,ti OR 'nivolumab':ab,ti OR 'pembrolizumab':ab,ti OR 'ipilimumab':ab,ti OR 'avelumab':ab,ti OR 'atezolizumab':ab,ti OR 'toripalimab':ab,ti OR 'benmelstobart':ab,ti OR 'anti-pd-1':ab,ti OR 'anti-pd-l1':ab,ti | ***96603*** |
| ***#3*** | 'tyrosine kinase inhibitor':ab,ti OR 'tki':ab,ti OR 'axitinib':ab,ti OR 'cabozantinib':ab,ti OR 'lenvatinib':ab,ti OR 'sunitinib':ab,ti OR 'anlotinib':ab,ti | ***80061*** |
| ***#4*** | 'randomized':ab,ti OR 'randomised':ab,ti OR 'phase iii':ab,ti OR 'phase 3':ab,ti OR 'rct':ab,ti | ***1660536*** |
| ***#5*** | *#1 AND #2 AND #3 AND #4* | ***Final search***  ***887*** |
| ***Cochrane Library*** | | |
| ***#1*** | (Carcinoma, Renal Cell OR renal cell carcinoma OR kidney cancer OR RCC OR metastatic renal cell carcinoma OR advanced renal cell carcinoma OR mRCC):ti,ab OR MeSH descriptor:[Carcinoma, Renal Cell] explode all trees | ***7165*** |
| ***#2*** | (Protein Kinase Inhibitors OR tyrosine kinase inhibitor OR TKI OR axitinib OR sunitinib OR cabozantinib OR lenvatinib OR anlotinib):ti,ab OR MeSH descriptor:[Protein Kinase Inhibitors] explode all trees | ***8769*** |
| ***#3*** | (immune checkpoint inhibitor OR nivolumab OR pembrolizumab OR ipilimumab OR avelumab OR atezolizumab OR toripalimab OR benmelstobart OR anti-PD-1 OR anti-PD-L1):ti,ab OR MeSH descriptor:[Immune Checkpoint Inhibitors] explode all trees | ***15129*** |
| ***#4*** | (Randomized Controlled Trial OR randomized OR randomised OR "phase III" OR "phase 3" OR RCT):ti,ab OR MeSH descriptor:[Randomized Controlled Trial] explode all trees | ***1156444*** |
| ***#5*** | *#1 AND #2 AND #3 AND #4* | ***Final search***  ***489*** |
| ***Web of Science*** | | |
| ***#1*** | TS=((renal cell carcinoma）OR（kidney cancer）OR（metastatic renal cell carcinoma）OR（mRCC)) | ***120535*** |
| ***#2*** | TS=((immune checkpoint inhibitor）OR(nivolumab）OR(pembrolizumab）OR(ipilimumab）OR(avelumab）OR(atezolizumab）OR(toripalimab）OR(benmelstobart）OR(anti-PD-1）OR(anti-PD-L1)) | ***102186*** |
| ***#3*** | TS=((tyrosine kinase inhibitor）OR(TKI）OR(axitinib）OR(cabozantinib）OR(lenvatinib）OR(sunitinib）OR(anlotinib)) | ***111173*** |
| ***#4*** | TS=((randomized）OR(randomised）OR(phase III）OR(phase 3）OR(RCT)) | ***2193390*** |
| ***#5*** | *#1 AND #2 AND #3 AND #4* | ***Final search***  ***811*** |

**Supplementary Figure 1: Risk of bias summary**


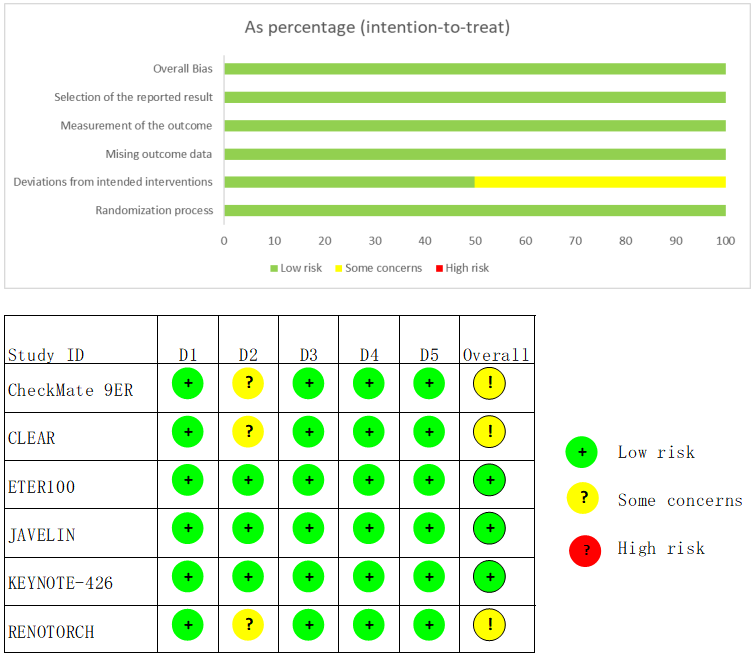


**Notes:** (**A**): Bar chart comparing the percentage risk of bias for each included RCT. Low risk of bias (Green), high risk of bias (Red), and unclear risk of bias (Yellow). (**B**): Risk of bias for each included RCT across five assessed domains (D1: Randomization process, D2: Deviations from intended interventions, D3: Missing outcome data, D4: Measurement of the outcome, D5: Selection of the reported result) and overall.

**Supplementary Table 3: Summary of findings and evidence certainty assessment (GRADE)**

| **Patient or population:** adults with previously untreated advanced/metastatic renal cell carcinoma **Intervention:** immune-checkpoint inhibitor plus tyrosine-kinase inhibitor (ICI + TKI) **Comparison:** tyrosine-kinase inhibitor alone (sunitinib) **Outcome:** progression-free survival,overall survival,objective response rate and grade ≥ 3 TRAEs | | | | | | |
| --- | --- | --- | --- | --- | --- | --- |
| Outcome | Studies (n) | Participants (n) | Effect measure (95% CI) | Control event rate | Absolute effect (per 1,000) | Certainty (GRADE) |
| Progression-free survival (PFS) | 5 | 3637 | HR 0.58 (0.51-0.67) | 2y: 335/1000 | 141 fewer per 1000 (111 fewer to 164 fewer) | ⊕⊕⊕⊕ High |
| Overall survival (OS) | 5 | 3637 | HR 0.79 (0.72-0.87) | 2y: 774/1000 | 163 fewer per 1000 (101 fewer to 217 fewer) | ⊕⊕⊕⊕ High |
| Objective response rate (ORR) | 5 | 3637 | OR 3.74 (2.62-5.32) | 329/1000 | 429 more per 1000 (297 more to 513 more) | ⊕⊕⊕⊕ High |
| Grade ≥ 3 TRAEs | 5 | 3587 | RR 1.12 (1.04-1.20) | 633/1000 | 76 more per 1000 (25 more to 127 more) | ⊕⊕⊕⊕ High |
| **TRAEs:** treatment-related adverse events; **HR:** hazard Ratio; **OR:** odds Ratio; **RR:** risk ratio. | | | | | | |
| GRADE Working Group grades of evidence **High certainty:** we are very confident that the true effect lies close to that of the estimate of the effect. **Moderate certainty:** we are moderately confident in the effect estimate; the true effect is likely to be close to the estimate of the effect, but there is a possibility that it is substantially different. **Low certainty:** our confidence in the effect estimate is limited; the true effect may be substantially different from the estimate of the effect. **Very low certainty:** we have very little confidence in the effect estimate; the true effect is likely to be substantially different from the estimate of effect. | | | | | | |

**Supplementary Table 4: Characteristics of the included studies**

| Study and setting | Study Design | Sample Size | | Age,median (range) | | Gender (M/F) | | Risk population | follow-up (months) | Interventions | |
| --- | --- | --- | --- | --- | --- | --- | --- | --- | --- | --- | --- |
|  |  | T | C | T | C | T | C |  |  | T | C |
| CheckMate 9ER Motzer et al. 2026 | Phase III | 323 | 328 | 62 (29-90) | 61 (28-86) | 249/74 | 232/96 | All risk | 67.6 | Nivolumab 240 mg +Cabozantinib 40 mg | Sunitinib |
| CLEAR  Motzer et al. 2024 | Phase III | 355 | 357 | 64 (34-88) | 61 (29-82) | 255/100 | 275/82 | All risk | 49.8 | Pambolizumab 200 mg +Lenvatinib 20 mg | Sunitinib |
| ETER100 Zhou et al. 2025 | Phase III | 264 | 263 | 60 (54-67) | 59 (54-67) | 204/60 | 196/67 | All risk | 22.8 | Benmelstobart 1200 mg + Anlotinib 12 mg | Sunitinib |
| JAVELIN Renal 101 Choueiri et al. 2025 | Phase III | 442 | 444 | 62 (29-83) | 61 (27-88) | 316/126 | 344/100 | All risk | 73.7 | Avelumab 10 mg/kg +Axitinib 5 mg | Sunitinib |
| KEYNOTE-426 Plimack et al. 2023 | Phase III | 432 | 429 | 62 (30-89) | 61 (26-90) | 308/124 | 320/109 | All risk | 67.2 | Pambolizumab 200 mg +Axitinib 5 mg | Sunitinib |
| RENOTORCH Yan et al. 2024 | Phase III | 210 | 211 | 60 (20-78) | 60 (28-78) | 162/48 | 157/54 | Intermediate/Poor  population | 14.6 | Toripalimab 240mg+Axitinib 5mg | Sunitinib |

**Supplementary Figure 2: Sensitivity analysis for objective response rate**


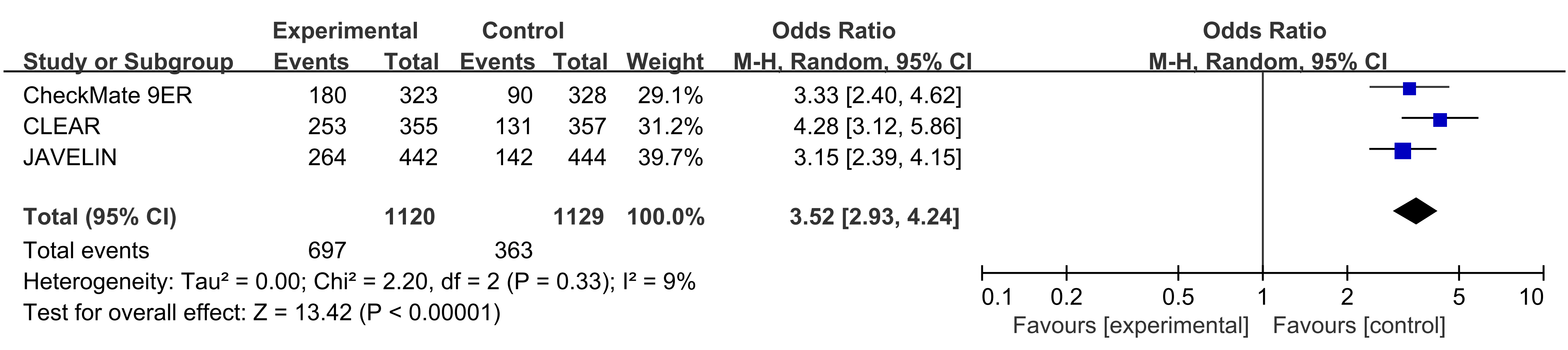


**Supplementary Figure 3: Sensitivity analysis for the risk of any-grade TRAEs**

**
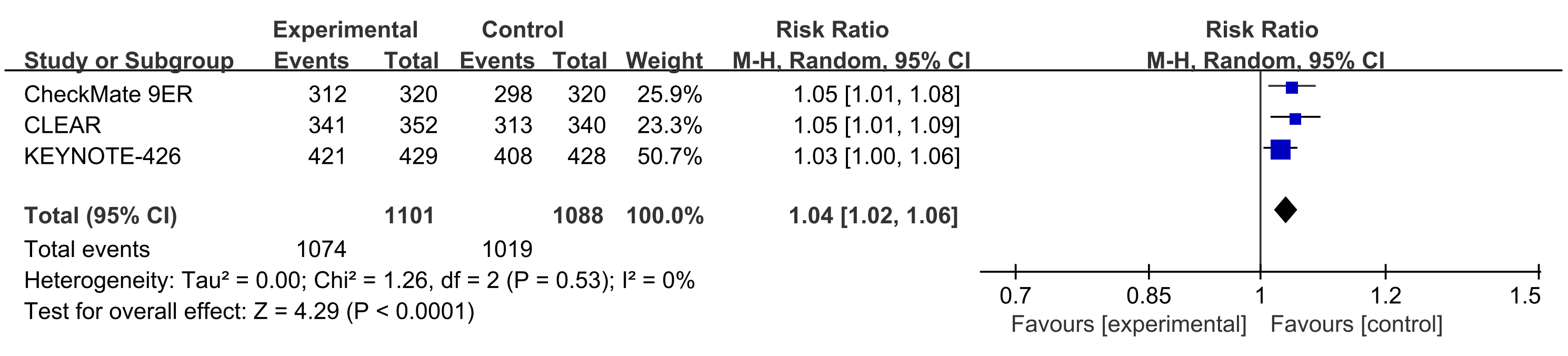
**

**Supplementary Figure 4: Sensitivity analysis for the risk of grade ≥ 3 TRAEs**

**
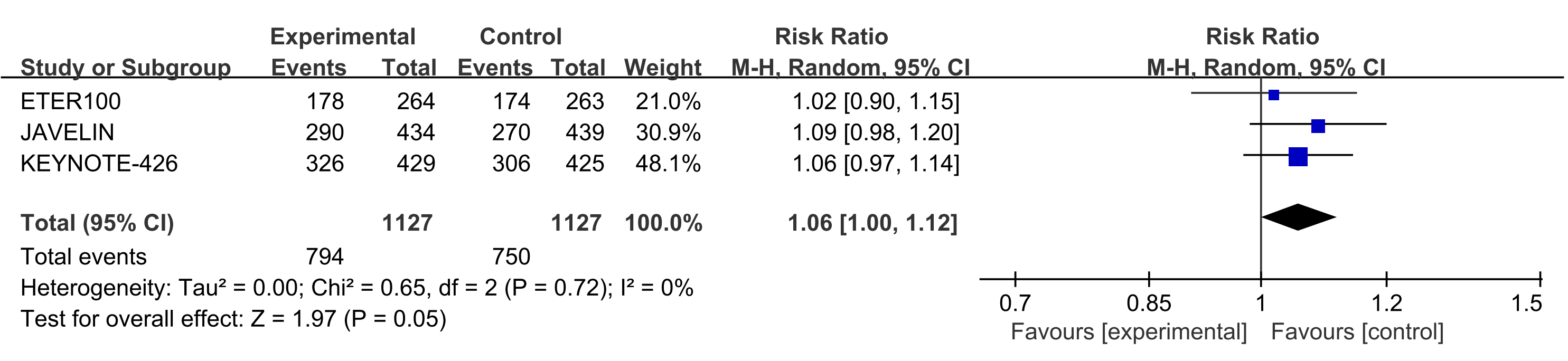
**
